# Is Bax/Bcl-2 Ratio Considered as a Prognostic Marker with Age and Tumor Location in Colorectal Cancer? 

**DOI:** 10.6091/ibj.1366.2015

**Published:** 2015-04

**Authors:** Ehsan Khodapasand, Narges Jafarzadeh, Farid Farrokhi, Behnam Kamalidehghan, Massoud Houshmand

**Affiliations:** 1 *Dept. of Research and Education, Razavi Hospital, Mashhad, Iran; *; 2 *National Institute for Genetic Engineering and Biotechnology, Tehran, Iran; *; 3 *Dept. of Pharmacy, Faculty of Medicine, University of Malaya, 50603 Kuala Lumpur, Malaysia*

**Keywords:** Colorectal cancer, Bax/Bcl-2 ratio, Bax expression, Bcl-2 expression

## Abstract

**Background:**

Bax and Bcl-2 are the major members of Bcl-2 family whose play a key role in tumor progression or inhibition of intrinsic apoptotic pathway triggered by mitochondrial dysfunction. Therefore, the balance between pro- and anti-apoptotic members of this family can determine the cellular fate.

**Methods:**

In this study, the relative level of mRNA expression of Bax and Bcl-2 genes was determined using RNA extraction, cDNA synthesis and RT-qPCR technique from 22 tumoral tissues and adjacent non-tumoral tissues from adenocarcinoma colorectal cancer.

**Results:**

The potential prognostic and predictive significance of Bax and Bcl-2 gene expression and Bax/Bcl-2 ratio were demonstrated in colorectal cancer. The significant correlation between qPCR data and different clinicopathologic parameters of colorectal carcinoma, including age, gender, tumor size, tumor stage, tumor location, and tumor differentiation was also examined. Interestingly, no significant correlation was seen between Bax and Bcl-2 expressions and clinicopathological parameters of colorectal cancer. However, Bax/Bcl-2 ratio was statistically correlated with age and tumor location. Patients with age above 50 showed decreased levels of Bax/Bcl-2 ratio. Moreover, the Bax/Bcl-2 ratio was significantly lower in tumors resected from colon compared to sigmoid colon, rectosigmoid and rectum tumors.

**Conclusion:**

This study indicates a significant correlation between age and tumor location with Bax/Bcl-2 expression ratio, suggesting predictive value as a potential molecular marker of colorectal cancer.

## INTRODUCTION

Colorectal cancer is the third most common malignancy and the fourth common cause of cancer death across the world [1, 2]. A tendency for the annual increase in the incidence of the colorectal cancer has been observed [3]. Inhibition of apoptosis is a fundamental element in carcinogenesis of colorectal cancer and also other human malignancies [4, 5]. The ability of cancer cells to escape from apoptosis, which abolishes cells with harmful genetic defects, can transform these cells into fatal immortal colonies of cells, which are well known as tumors.

Bcl-2 family has been discovered to play a key role in promoting or inhibiting intrinsic apoptotic pathway triggered by mitochondrial dysfunction [6, 7]. Therefore, the balance between pro- and anti-apoptotic members of this family can determine the cellular fate. Bax and Bcl-2 are the major members of Bcl-2 family whose potential roles in tumor progression and prognosis of different human malignancies have been of interest in various studies during the last decade. Bax promotes cell death through permeabilization of mitochondrial outer membrane in response to different cellular stresses. In contrast, Bcl-2 prevents apoptosis by inhibiting the activity of Bax [7, 8]. It has been demonstrated that the absence of Bax expression in colorectal cancer cells can induce resistance to apoptosis triggered by different chemotherapeutic agents [9-11]. Conflicting results have been reported in the case of introducing Bax expression as an independent prognostic and predictive marker of colorectal cancer. Apparently, a significant correlation between longer survival and increased Bax expression in tumor cells has been observed in previous studies [12, 13]. However, in other studies, down-regulation and up-regulation of Bax and Bcl2 expressions were associated with better survival, respectively [13, 14]. These results became more confusing when therapeutic status of patients was considered [15]. It has been shown that in a surgery-alone group of patients, high Bax expression is associated with improved survival [16]. However, patients with lack or low Bax expression in their tumor cells, but not those with high expression, benefit from 5-FU-based adjuvant therapies. On the other hand, the role of Bcl-2 in development or progression of colorectal carcinoma and prognosis of the disease has not been fully elucidated [14].

The aim of the study was to determine the relative level of mRNA expression of Bax and Bcl-2 genes and Bax/Bcl-2 ratio in tumoral tissues and adjacent non-tumoral tissues, and to evaluate the correlation between Bax/Bcl-2 ratio and clinicopathological parameters of colorectal carcinoma.

## MATERIALS AND METHODS

***Ethical statement and Sample collection.*** Ethics approval and patients' informed consent, including consent to participate in the study and consent to publish were obtained for the present study in accordance to the Razavi Hospital and Medical Ethics Committee of Razavi Hospital (Approval No. RH.1018), Mashhad, Iran. A number of 22 patients with colorectal adenocarcinoma at various stage of progression were included in this study (Table 1). There were 15 males and 7 females with a median age of 50 years (range 16-84 years). Surgically removed tumoral tissues and adjacent non-tumoral tissues were collected and stored in RNAlater stabilization reagent (Qiagen, Germany) at 4ºC, in which 14 and 8 tumors were removed from the rectum and colon, respectively.

***RNA extraction and cDNA synthesis. ***Total RNA was extracted from tumoral tissues and adjacent non-tumoral tissues using the RNeasy mini kit (Qiagen, Germany) according to the manufacturer’s instruction. The integrity of the extracted RNA was confirmed by electrophoresis on 1% agarose gel. The concentration and purity of RNA was identified using spectro-photometer (Amersham bioscience, UK). The cDNA was synthesized using Revert-aid first strand cDNA synthesis kit (Fermentas, Thermo Fisher Scientific, USA) using random hexamer primer according to the manufacturer’s instruction in 20-µl reaction mixture.

***Primer design. ***Primers were designed using Primer3 software according to the published gene sequence in the Bax and Bcl-2 Gene Bank as reported (Table 2) in the literature and synthesized by TAG Company (Copenhagen, Denmark). Primer sequence homology and total gene specificity were confirmed with BLAST analysis (www.ncbi.nlm.nih.gov/blast). Each primer pair recognized at least one exon/intron junction to avoid amplification of genomic DNA.

**Table 1:** Clinical characteristics of 22 patients with colorectal adenocarcinoma

**Patient no**.	**Age**	**Sex**	**Site of primary**	**Tumor size (cm)**	**Grade**	**Stage**
A00209	59	F	RECT	5.0	I	2a
A00360	34	M	RECT	3.0	I	1
A00422	52	M	RECT	6.0	II	3b
A00585	60	F	Rectosigmoid	2.5	I	3b
A00883	27	M	Rectosigmoid	4.5	II	4
A00933	53	M	Rectosigmoid	5.0	I	4
A00948	48	M	RECT	4.0	II	2a
A00997	55	F	RECT	5.0	I	2a
A01118	62	M	Colon, NOS	4.0	I	2a
A01180	19	M	RECT	5.0	III	3b
A01187	16	M	Sigmoid Colon	7.0	III	3b
A01237	48	M	Colon, NOS	6.0	III	3c
A01293	64	M	Rectosigmoid	4.0	I	3c
A01294	67	M	Rectum	2.5	II	3c
A01297	56	F	Sigmoid Colon	8.0	I	2a
A01303	63	M	Rectum	6.0	III	3c
A01357	84	F	Colon, NOS	10.0	II	2a
A01361	80	F	Sigmoid Colon	6.5	I	3b
A01384	50	F	Rectum	3.5	I	2a
A01387	46	M	Rectosigmoid	4.5	II	2a
A01447	66	M	Colon, NOS	3.0	I	1
A01448	55	M	Sigmoid Colon	3.6	I	3

F, female; M, male; NOS, not otherwise specified; RECT, rectosigmoid

**Table 2:** Primer sequences used for QRT-PCR

**Primer**	**Sequences**	**T**_a_
Bax	Forward: 5’-CCTGTGCACCAAGGTGCCGGAACT-3’ Reverse: 5’-CCACCCTGGTCTTGGATCCAGCCC-3’	59ºC
Bcl-2	Forward: 5’-TTGTGGCCTTCTTTGAGTTCGGTG-3’ Reverse: 5’-GGTGCCGGTTCAGGTACTCAGTCA-3’	59ºC
β-actin	Forward: 5’-GGCGGCACCACCATGTACCCT-3’ Reverse: 5’-AGGGGCCGGACTCGTCATACT-3’	59ºC

***Quantitative RT-PCR. ***Relative expression of Bax and Bcl-2 genes was performed using 2 µl synthesized cDNA (1,600 ng/µl) as the template in 12.5 µl SYBR Green PCR Master Mix and 2 µl each primer using Rotor-Gene 6000 real-time rotary analyzer (Corbett Life Science, USA). Then, the following conditions were selected: 95ºC-10 min, 45 cycles: 95º-30 s, 59ºC-30 s, and 72º-45 s. β-actin was used as endogenous control gene, and all experiments were performed in triplicate for each data point. The specificity of qPCR reaction was confirmed by melt curve analysis and electrophoresis of PCR products on 1.5% agarose gel. Standard curves for each of β-actin, Bax, and Bcl-2 genes were generated by amplifying 5× serial dilutions of cDNA created from RNA isolated from normal tissue. Two standard curve methods (Rotor-Gene 6000 series software, v1.7) were used for relative quantitation of Bax and Bcl-2 mRNA expression (Copies/µl), and the triplicate samples were assessed for variability using geometric standard deviations. The geometric mean of the triplicate run for each gene of interest was normalized with the geometric mean of *β-actin.*

***Statistical analysis. ***All statistical analyses were performed using SPSS v.16 (IBM, Chicago, IL, USA). Correlations between relative level of expression of Bax and Bcl-2 genes and age, tumor stage, and tumor size of the patients were analyzed using Pearson's correlation coefficient. *t*-test was also used to analyze the significance of patient’s gender. Furthermore, correlation of expression rates with remaining parameters, including tumor location and differenti-ation was performed using one-way ANOVA, followed by a Tukey's post hoc test. Differences between Bax/Bcl-2 ratio in normal and tumoral tissue were also analyzed using paired *t*-test. *P *value < 0.05 was considered statistically significant.

## RESULTS AND DISCUSSION

**Fig. 1:**
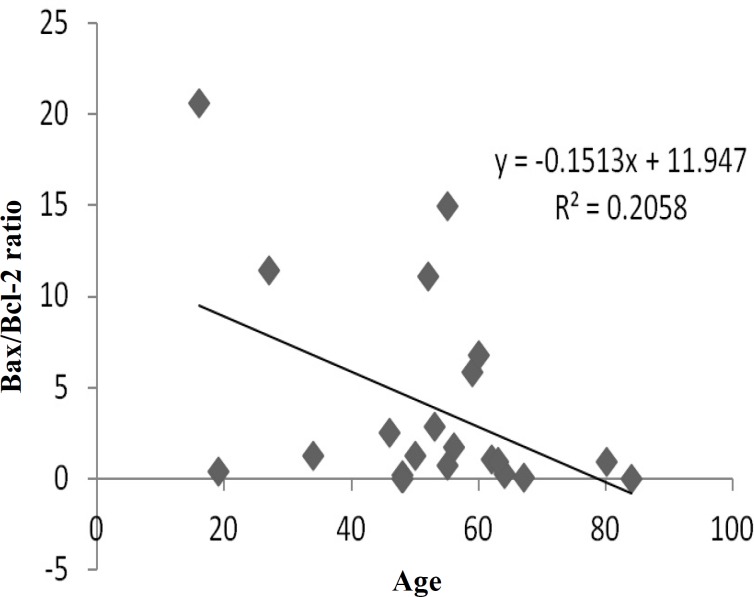
Correlation of Bax/Bcl-2 ratio with patient’s age. The ratio decreased in patients with age above 50, indicating an inverse correlation between Bax/Bcl-2 ratio and age.

**Fig. 2:**
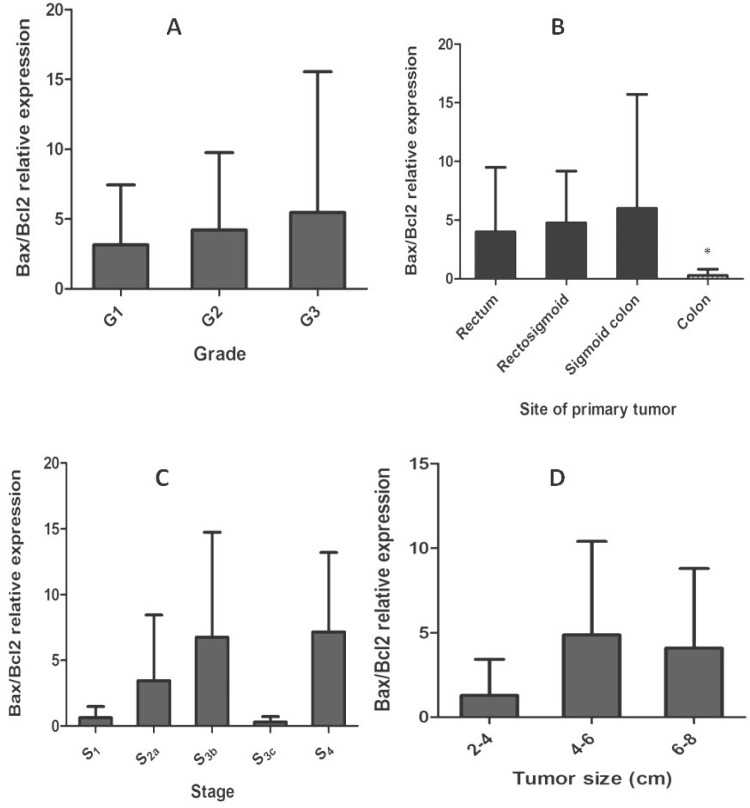
Bax/Bcl-2 relative expression in colorectal cancer cells with grade, site of primary tumor, stage, and tumor size. Panel (A) shows Bax/Bcl-2 expression ratio increases slightly from well-moderately to poorly differentiated tumors (*P*>0.05). Panel (B) indicates the values of Bax/Bcl-2 ratio in four different groups of primary tumor sites (mean ± SD). The ratio was significantly lower in tumors resected from colon (*P *value: 0.020). *Statistically significant *P* < 0.05. Panel (C) shows Bax/Bcl-2 relative expression in function of staging of colorectal carcinoma. This ratio was not significantly correlated with different stages of colorectal tumors (*P* > 0.05). Panel (D) indicates Bax/Bcl-2 relative expression with different tumor sizes ranged approximately from 2 to 8 cm in size. Bax/Bcl-2 ratio was lower in smaller tumors, but a strong correlation was not observed. ^*^Statistically significant *P *< 0.05

Dysregulation of the mitochondrial pathway of apoptosis is one of the most important events during carcinogenesis. Bcl-2 protein family, including anti-apoptotic (e.g. Bcl-2, Bcl-xl, and Mcl-1) and pro-apoptotic (e.g. Bax, Bak) members play a central role in regulation of this pathway [17]. Relative level of mRNA expression of Bax and Bcl-2 genes and Bax/Bcl-2 ratio was evaluated in tumoral and adjacent non-tumoral tissues of colorectal cancer patients. Regarding statistical analysis, no significant correlation was observed between relative level expression of Bax and Bcl-2 with different clinicopathological parameters, including age, gender, tumor size, tumor location, tumor stage, and tumor differentiation (Table 3). According to our results, Bax and Bcl-2 expression was not significantly correlated with patient's gender and age less than 50, size, stage (I-IV), differentiation status, and location of primary tumor which could be colon, sigmoid colon, rectosigmoid, and rectum (*P *> 0.05). However, Bax/Bcl-2 ratio was significantly (*P* < 0.05) correlated with patient’s age above 50 (Fig. 1), and site of primary tumor in colon (Fig. 2), in comparison to other sites, including sigmoid colon, rectosigmoid, and rectum (*P* > 0.05).

**Table 3:** Correlation between the expression of Bax and Bcl-2 with clinicopathological features of patients with colorectal cancer

**Characteristics**	**No (%)**	**Relative Bax expression (tumor/normal) (no. %**)				**Relative Bcl-2 expression (tumor/normal) (no %)**				**Bax/Bcl-2 ratio (No %)**		
		**>1**	**<1**	***P value***		**>1**	**<1**	***P value***		**>1**	**<1**	***P value***
Gender Male Female	15 (68.2) 7 (31.8)	10 (66.7) 5 (71.4)	5 (33.3) 2 (28.6)	0.581		11 (73.3) 6 (85.7)	4 (26.7) 1 (14.3)	0.883		7 (46.7) 5 (71.4)	8 (53.3) 2 (28.6)	0.917
												
Age <50 ≥50	7 (31.8) 15 (68.2)	5 (71.4) 10 (66.7)	2 (28.6) 5 (33.3)	0.148		6 (85.7) 10 (66.7)	1 (14.3) 5 (33.3)	0.199		4 (57.1) 8 (53.3)	3 (42.9) 7 (46.7)	0.049*
												
Site of primary tumor Colon Rectum	8 (36.4) 14 (63.6)	3 (37.5) 12 (85.7)	5 (62.5) 2 (14.3)	0.126		6 (75.0) 10 (71.4)	2 (25.0) 4 (28.6)	0.513		4 (50.0) 9 (64.3)	4 (50.0) 5 (35.7)	0.020*
												
Tumor differentiation Well Moderate Poor	12 (54.5) 6 (27.3) 4 (18.2)	9 (75.0) 4 (66.7) 2 (50.0)	3 (25.0) 2 (33.3) 2 (50.0)	0.852		8 (66.7) 5 (83.3) 3 (75.0)	4 (33.3) 1 (16.7) 1 (25.0)	0.663		9 (75.0) 3 (50.0) 1 (25.0)	3 (25.0) 3 (50.0) 3 (75.0)	0.428
												
Tumor size <4.8 ≥4.8	11 (50.0) 11 (50.0)	10 (90.9) 7 (63.6)	1 (9.1) 4 (36.7)	0.778		9 (81.8) 7 (63.6)	2 (18.2) 4 (36.7)	0.424		7 (63.6) 6 (54.5)	4 (36.7) 5 (45.5)	0.187
												
Tumor stage I II III IV	2 (9.1) 8 (36.4) 10 (45.4) 2 (9.1)	1 (50.0) 5 (62.5) 7 (70.0) 2 (100.0)	1 (50.0) 3 (37.5) 3 (30.0) 0 (0)	0.794		1 (50.0) 6 (75.0) 7 (70.0) 1 (50.0)	1 (50.0) 2 (25.0) 3 (30.0) 1 (50.0)	0.906		1 (50.0) 6 (75.0) 3 (30.0) 2 (100.0)	1 (50.0) 2 (25.0) 7 (70.0) 0 (0.0)	0.818

As indicated in previous studies, expression of Bax and Bcl-2 proteins may be helpful in predicting clinical outcome [14, 18], patient’s survival [12], and even response to chemotherapeutic agents in colorectal carcinoma [9, 15]. We analyzed the relative expression of these two proteins at mRNA level in a heterogeneous group of patients with colorectal carcinoma using qPCR technology.

There are increasing evidences trying to prove the prognostic and predictive role of apoptosis-related markers such as Bax and Bcl-2. Sturm *et al. *[12] reported that in patients with hepatic metastasis of colorectal cancer, high Bax expression was correlated with longer survival, especially in patients with wild-type p53. In accordance with previous studies, Katkoori *et al. *[15] reported that in the surgery-alone group, high Bax expression was associated with better survival. They showed that in a group of patients who underwent 5-FU-based adjuvant chemotherapy, low Bax expression was associated with improved survival, suggesting that there are apparently distinct mechanisms of Bax involvement in the manifestation of apoptosis triggered by chemotherapeutic agents. Interestingly, various investigations have shown that in colorectal cancer cells lacking Bax, apoptosis can be partially or completely abolished in response to chemotherapeutic agents such as 5-FU and sulindac [9-11]. Jansson and Sun [18] have demonstrated that Bax expression is decreased from primary to metastatic and also from well/moderately to poorly differentiated tumors. Furthermore, in this study, the cases with decreased Bax expression from primary tumors to the corresponding metastases showed a more infiltrative growth pattern, more distant metastases, and a weak trend toward poor prognosis, indicating the involvement of Bax expression in tumor differentiation and metastatic progression.

Although lots of studies have been done on the prognostic significance of counteracting twin of Bcl-2 and Bax, most of them failed to find a significant relationship between Bcl-2 expression levels and clinicopathological parameters of colorectal cancer. A possible explanation for these results can be the presence of other members of Bcl-2 family which can act independently from Bcl-2 [3, 11, 19, 20]. Bax/Bcl-2 ratio can act as a rheostat which determines cell susceptibility to apoptosis [21]. Lower levels of this ratio may lead to resistance of human cancer cells to apoptosis. Thus, Bax/Bcl-2 ratio can affect tumor progression and aggressiveness. Our results are in line with a previous study which suggested that low levels of Bax/Bcl-2 ratio may result in poor prognosis and more infiltrative growth pattern in older patients [22]. On the other hand, it has been demonstrated that location of primary tumor has a critical role in determining tumor characteristics and prognosis of the cancer [23, 24]. Our findings remarked the importance of location of primary tumor and indicated that tumors resected from colon have different gene expression profile especially in the case of Bax and Bcl-2, which can affect tumor characteristics. These tumors seem to be more aggressive compared to tumors located in the other sites.

In accordance with a previous study [25], relative level of expression of Bax and Bcl-2 failed to reach statistical significance. Paradoxical behavior of tumor cells in expressing different oncogenes and tumor suppressor genes, which has been reported in various studies [18, 26], and also dysregulation of many intracellular signaling pathways, which make them difficult to understand, might be accounted for this finding. However, there is a stronger possibility that different chemotherapeutic agents may affect the expression level and clinical roles of molecules, which involve in apoptosis, especially Bax and Bcl-2 [15]. In this study, a group of patients independent of their therapeutic status was analyzed. Therefore, their possible chemotherapy either radiotherapy regimens may have altered the expression levels of Bax and Bcl-2 proteins. Prognostic and predictive role of Bcl-xl gene expression in colorectal cancer has also been reported by some researchers [27, 28]. Moreover, expression analysis of BH3-only members, a group of small molecules with pro-apoptotic activity, may be helpful in elucidating alterations in Bax and Bcl-2 expression level in human colorectal carcinoma [8].

In conclusion, Bax and Bcl-2 expression was most predictive of outcome when combined as Bax/Bcl-2 expression ratio in colorectal tumors compared to expression levels of Bax and/or Bcl-2 genes alone. Moreover, further analyses on larger homogeneous cohorts of patients with similar therapeutic status and also tumor characteristics such as tumor stage and differentiation may be very useful from different aspects, including prognosis of the disease, predicting patient’s survival, tumor relapse, and also response to chemotherapeutic agents.
